# Acute NMDA toxicity in cultured rat cerebellar granule neurons is accompanied by autophagy induction and late onset autophagic cell death phenotype

**DOI:** 10.1186/1471-2202-11-21

**Published:** 2010-02-18

**Authors:** Shankar Sadasivan, Zhiqun Zhang, Stephen F Larner, Ming C Liu, Wenrong Zheng, Firas H Kobeissy, Ronald L Hayes, Kevin KW Wang

**Affiliations:** 1 Center for Neuroproteomics and Biomarkers Research, Department of Psychiatry, the McKnight Brain Institute of the University of Florida, Gainesville, FL 32610, USA; 2 Center of Innovative Research, Banyan Biomarkers, Inc, Alachua, FL 32615, USA

## Abstract

**Background:**

Autophagy, an intracellular response to stress, is characterized by double membrane cytosolic vesicles called autophagosomes. Prolonged autophagy is known to result in autophagic (Type II) cell death. This study examined the potential role of an autophagic response in cultured cerebellar granule neurons challenged with excitotoxin N-methyl-D-aspartate (NMDA).

**Results:**

NMDA exposure induced light chain-3 (LC-3)-immunopositive and monodansylcadaverine (MDC) fluorescent dye-labeled autophagosome formation in both cell bodies and neurites as early as 3 hours post-treatment. Elevated levels of Beclin-1 and the autophagosome-targeting LC3-II were also observed following NMDA exposure. Prolonged exposure of the cultures to NMDA (8-24 h) generated MDC-, LC3-positive autophagosomal bodies, concomitant with the neurodegenerative phase of NMDA challenge. Lysosomal inhibition studies also suggest that NMDA-treatment diverted the autophagosome-associated LC3-II from the normal lysosomal degradation pathway. Autophagy inhibitor 3-methyladenine significantly reduced NMDA-induced LC3-II/LC3-I ratio increase, accumulation of autophagosomes, and suppressed NMDA-mediated neuronal death. ATG7 siRNA studies also showed neuroprotective effects following NMDA treatment.

**Conclusions:**

Collectively, this study shows that autophagy machinery is robustly induced in cultured neurons subjected to prolonged exposure to excitotoxin, while autophagosome clearance by lysosomal pathway might be impaired. Our data further show that prolonged autophagy contributes to cell death in NMDA-mediated excitotoxicity.

## Background

Autophagy is an intracellular pathway that is activated in response to cell stress. It is a phenomenon where the cytoplasmic organelles in the cell are engulfed by double membrane vesicles called the autophagosomes and delivered to the lysosomes where the organelles are broken down by lysosomal proteases and the amino acids recycled back into the cell machinery to aid cell survival [1,2]. Some of the key autophagy protein (Atg) identified to be involved in this process are Atg4, Atg6, Atg8, Atg12 and Atg5 [3]. Autophagy has been reported to be vital in the development of the central nervous system [4,5]. It has also been documented to be constitutively active in the healthy neurons and aid survival [6]. Researchers have used a number of tools to study and interpret autophagy induction [7]. For example, an elevated level of Bcl-2-binding protein Beclin-1 (Atg6) has been documented to be indicative of autophagy induction. Another protein marker for autophagy induction extensively studied, is the lipidated form of microtubule associated protein light chain-3 (MAP-LC3) found on the outer and to a lesser extent the inner membrane of the double membrane of the autophagosome.

Programmed cell death among neurons in the central nervous system is a regulated process. Neurons undergo either apoptotic (type I) or autophagic (type II) cell death or oncotic/necrotic (type III) depending on the nature of insult [8,9]. Acute excitotoxic insults resulting from the use of glutamate in primary culture has been shown to induce both oncotic and apoptotic cell death in neurons [10,11]. Increased excitation of the glutamate receptors by its ligand has been shown to cause an imbalance in the ionic gradient in neurons, resulting in an increase in the calcium and sodium levels intracellularly leading to oncosis. At the same time, this excessive activation in neurons has also been demonstrated to activate the endonucleases, causing internucleosomal DNA fragmentation, thus resulting in apoptosis. Though extensive studies have been conducted on apoptotic cell death mechanisms, the biochemical mechanisms and the exact definition of "autophagic cell death" is poorly understood [12-16].

Autophagic vacuoles have been shown to accumulate in affected neurons of several neurodegenerative diseases such as Alzheimer's disease and Parkinson's disease. Wang et al., (2006) recently demonstrated that the induction of autophagy was associated with axonal degeneration in Purkinje cells in Lurcher mice. More recent experimental evidence has also shown the upregulation of autophagy protein Beclin-1 (Atg-6) and/or to LC3-II/LC3-I ratio increase in different rodent models of traumatic brain injury (TBI) [17-20].

Excitotoxicity via overactivation of ionotropic glutamate receptor subtype N-methyl-D-aspartate (NMDA)-receptor, is one of the documented hallmark events that occur following acute brain injury [21,22]. Hence we sought to examine if autophagy is a general response during excitotoxic NMDA challenge by using rat cerebellar granule neuronal (CGN) cultures *in vitro*. In addition, we hypothesize that autophagy and possible autophagic cell death might also participate in NMDA excitotoxicity.

## Results

### Acute NMDA exposure induces autophagy in cerebellar granule neurons in culture

**Figure 1:**
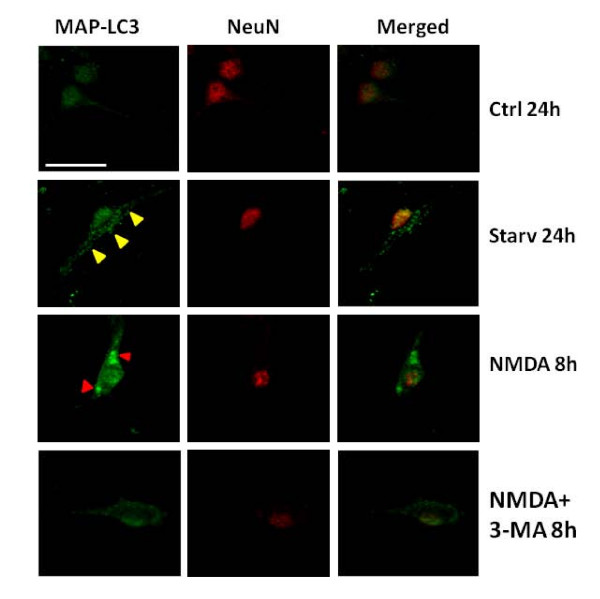
**NMDA excitotoxicity results in the induction of LC3-positive autophagosomes in rat cerebellar granule neurons**. Representative laser confocal fluorescent micrographs of cerebellar granule neurons in culture exposed to NMDA (200 μM) with or without co-treatment with 3-MA. NeuN (red) was used to stain mature neurons Arrowheads (yellow) represent the increased LC3 (green) staining of autophagosomes in the cell bodies of the neurons co-localized with neuronal marker NeuN (red). Red arrowheads represent the increased intensity of LC3 staining in the neuronal cell bodies at 8 hours. Neurons treated with serum free medium are labeled as controls (Ctrl) while neurons exposed to amino acid starvation conditions depict positive controls (Starv) for the induction of autophagy. All images were taken at 400× magnification. Scale bar represents 20 μm.

**Figure 2:**
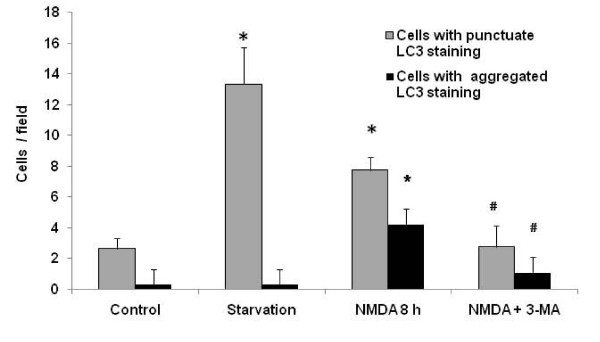
**Quantification of punctate stains in LC3-positive neurons**. Punctate stains were quantified in different microscopic fields (n = 4) and compared to neurons exhibiting densely stained LC3 immunopositive autophagosomes. The numbers were plotted and statistical analysis was performed using Student's t-test. *p < 0.05 denotes significant differences between different treatment groups to serum free medium treated control neurons (Ctrl). ^# ^p < 0.05 denotes statistical significance in treatment groups when compared to NMDA-treated neurons alone.

Rat cerebellar granule neurons (CGN) were treated with or without NMDA (200 μM) in serum free medium (SFM) to achieve excitotoxic and control conditions, respectively. To assess the possible induction of autophagy following acute NMDA exposure, the neurons were stained with an antibody against microtubule associated light chain-3 (LC3) protein, a known autophagy protein marker, also called Atg-8 [23]. Neurons subjected to NMDA exposure for 8 h exhibited increased number of both regular sized and unusually large LC3 immunopositive autophagosomes inside the neuronal cell body. Co-immunostaining with anti-NeuN antibody, a protein marker of mature neurons was employed to demonstrate that the increase in the LC3 positive autophagosomes was indeed found in neurons following NMDA treatment (Fig 1). The addition of autophagosome inhibitor 3-methyladenine (3-MA) to NMDA-treated CGN suppressed the increase of LC-3 positive staining. As a positive control, neurons subjected to amino acid starvation (24 hours) showed robust formation of punctuate LC-3 positive autophagosomes, when compared to untreated CGN. Acute exposure to NMDA results in an increase in neurons with anti-LC3-positive autophagosomes compared to control conditions, suggesting an inherent autophagy response to NMDA stress. This increase was quantified by counting the number of neurons demonstrating anti-LC3 positive regular sized autophagosomes as well as "unusually large" autophagosomal bodies (defined as at least 5-time the size of normal sized autophagosomes) (Fig 2).

**Figure 3:**
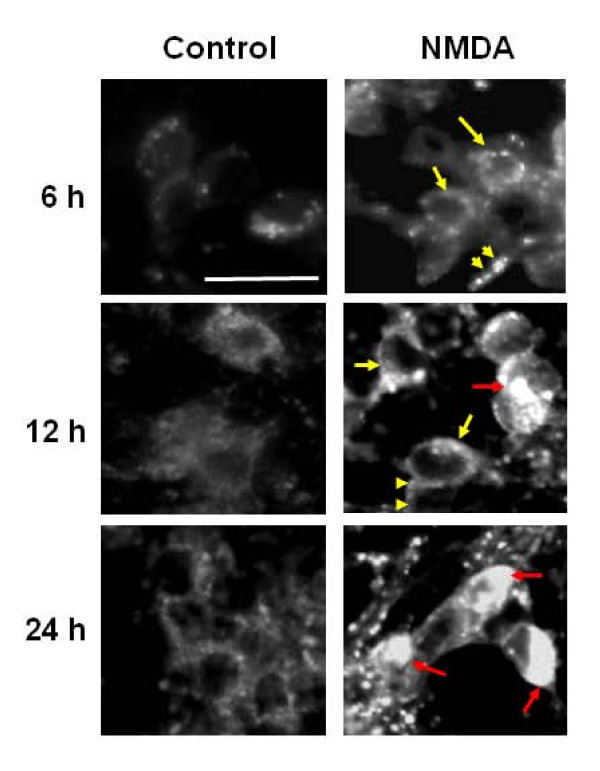
**NMDA exposure induces the formation of MDC-positive autophagosomes in cerebellar granule neurons**. Representative fluorescence micrographs of granule neurons incubated with monodansylcadaverine (MDC) show an increase in the labeling of the autophagosomes in both the neurites and the cell bodies. Arrows and arrow heads (yellow) indicate the normal punctate autophagosome staining at 6 and 12 hours in the cell bodies and neurites, respectively. Red arrows indicate the dense autophagosomes staining in the cell bodies following prolonged exposure to NMDA (12 to 24 hours). All images were taken at 400× magnification. Scale bar represents 10 μm.

Similarly, the signal from the fluorescent dye monodansylcadaverine (MDC) used to label acidic vesicles such as autophagosomes [24] also showed a strong increase in staining (yellow arrows) in both the cell bodies and the neurites in cell cultures 6 h after NMDA treatment (Fig 3).

### Autophagy protein markers beclin-1 and LC3-II are up-regulated following early phase of NMDA exposure

**Figure 4:**
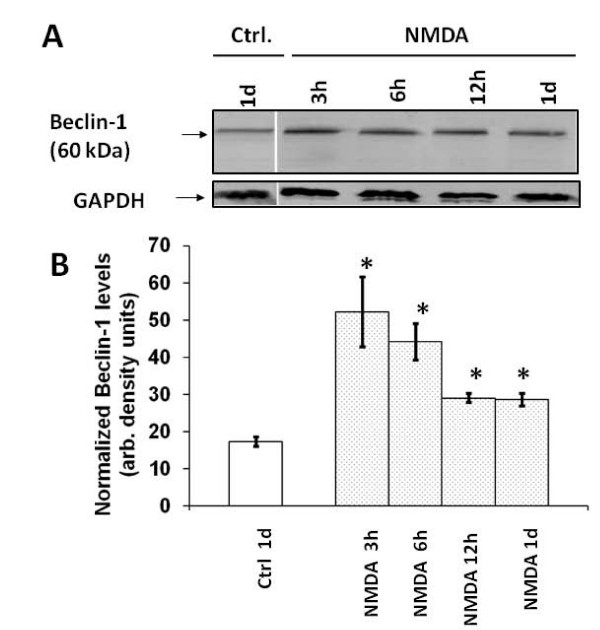
**NMDA exposure of neurons results in an increase in the beclin-1 levels *in vitro***. **A)** Lysates of neuronal cultures were obtained at time periods of 3, 6, 12 and 24 hours, treated with or without NMDA. These lysates were analyzed by immunoblots and probed with the anti-beclin-1 antibody (n = 3). **B) **Quantification of the autophagy protein beclin-1 bands in the immunoblots was plotted. The band intensities were normalized against the loading control. Increases in the band intensities of the beclin-1 levels were observed after NMDA-treated neuronal cultures as compared to controls. The expressed values are means ± S.E.M. (n = 3; *p < 0.05). GAPDH was used as a loading control.

Having established the induction of autophagy in neurons exposed to NMDA, we sought to study protein levels of the autophagy protein marker beclin-1 (Atg6). We performed immunoblots on cell lysates obtained from cultures following treatment with or without NMDA at different time periods. The beclin-1 levels appear to be increased in the NMDA-treated neurons when compared to controls at time periods ranging from 3 h to 24 h post treatment (Fig 4A). Densitometric quantification of Beclin-1 and normalization with GAPDH level in the same samples demonstrated significant increases in beclin-1 protein level at all time points after NMDA exposure when compared to controls (Fig 4B).

**Figure 5:**
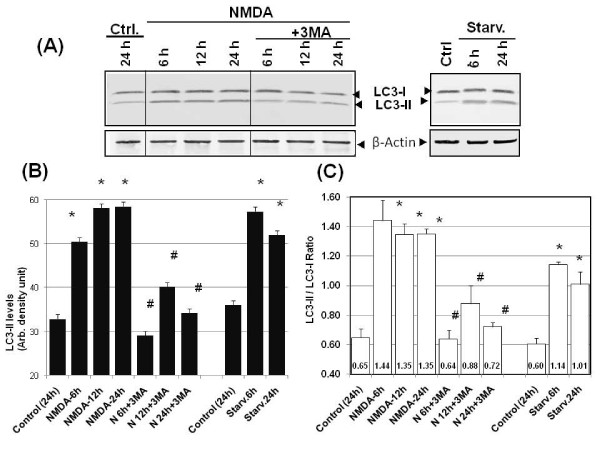
**NMDA challenge induced LC3-II accumulation which is 3-MA sensitive**. **A) **Lysates were obtained from control neuronal cultures (at 24 h) and at 6, 12 and 24 hours from neuronal cultures treated with NMDA or NMDA+3-MA (left panels). For classic autophagy positive controls, 6 h and 24 h amino acid starvation in neurons were also included (right panels). These lysates subjected to immunoblotting were probed with anti-LC3 and β-actin antibody. Representative immunoblots are shown (n = 4). β-actin was used as a loading control. **B) **LC3 immunoblot band intensities were quantified densitometrically and the values plotted. The expressed values are means ± S.E.M. (n = 4; *p < 0.05 compared to controls; #p < 0.05 compared to NMDA-treated). **C) **LC3-II/LC3-I ratio was also calculated plotted. The expressed values are means ± S.E.M. (n = 4); *NMDA groups or starvation groups significantly higher than control (*p < 0.05, Student paired T-test); ^#^3-MA treatment groups significantly lower than respective NMDA groups (^#^p < 0.05, Student paired t-test). The individual ratio value is also listed at the bottom of each bar.

In parallel, we also sought to examine if there was increased levels of the autophagosomes associated lipidated LC3-II form. Immunoblotting analysis of cell lysates from NMDA-exposed culture was performed using anti-LC3 antibody that detects both LC3-I and LC-3-II. In control CGN cells, we detected the presence of both LC3-I and LC3-II, in a LC3-II/LC3-I ratio of about 0.60-0.65, in favor of the larger form (Fig 5). The presence of endogenous levels of LC3-II here most likely represents the basal level of autophagy that exists in all resting cells. Upon NMDA treatment, importantly, a very rapid and robust increase of LC3-II was observed (Fig 5A and 5B). We had previously established that amino acid starvation could robustly induce autophagy [25]. Thus, amino acid starvation of CGN was also used here as positive controls. In fact, we observed an increase of LC3-II levels at 6 h and 24 h after starvation (Fig 5A and 5B). We also calculated LC3-II/LC3-I ratio after various time points of NMDA treatment (6 h, 12 h and 24 h) and they were 1.35-1.44, in favor of the lipidated form (Fig 5C). This ratio in fact compared favorably to those after starvation-induced autophagy in CGN (LC3-II/LC3-I ratio of 1.14 after 6 h starvation and 1.01 after 24 h starvation).

### Autophagy inhibitor 3-Methyladenine (3-MA) effectively suppresses NMDA-induced autophagy

**Figure 6:**
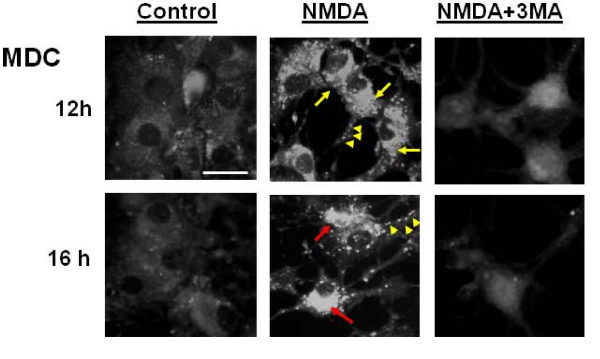
**NMDA-induced autophagosome formation is inhibited by 3-MA**. Representative fluorescence micrographs show an increase in Monodansylcadaverine (MDC; 0.05 mM) labeling of autophagosomes in neurons increase following acute exposure to NMDA (200 μM) compared to control conditions and co-treatment of NMDA+3-MA. Yellow arrows and yellow arrow heads indicate the presence of regularly sized autophagosomes in the cell bodies and neurites respectively in 12 and 16 hours after NMDA exposure. The red arrows in the image at 16 hours are indicative of the accumulation of the unusually large MDC-positive autophagosomes in the cell bodies of neurons following prolonged NMDA exposure. Images are taken at 400× magnification. Scale bar represents 20 μm.

Having observed NMDA-induced autophagy in CGN, we examined the autophagy inhibitor 3-methyladenine (3-MA, 10 mM) for its ability to suppress the process of autophagy under excitotoxic conditions. We investigated whether the addition of 3-MA would inhibit the increase of LC-3-II in NMDA-treated cells. Immunoblot data indeed demonstrated a reduction of the LC3-II levels (Fig 5). Consistent with the immunoblotting results, a reduction in autophagy induction was further confirmed using MDC staining. The MDC-positive autophagosomes were relatively sparse and weak in fluorescence intensity in CGN cultures co-treated with NMDA+3-MA when compared to cultures treated with NMDA (12 h) (Fig 6). We also noted that the intense MDC staining normally observed at 16 hours post-NMDA exposure was absent in the presence of 3-MA.

### Interaction between NMDA challenge and LC3-II flux and turnover

**Figure 7:**
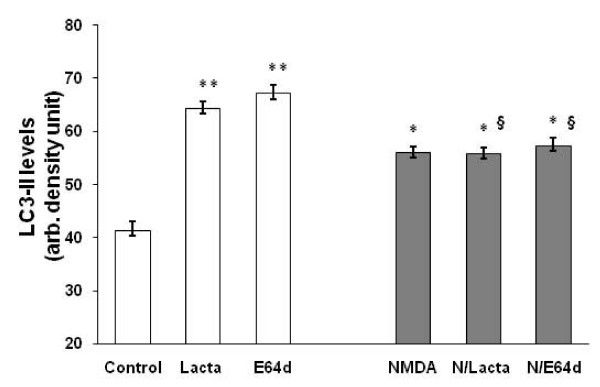
**NMDA challenge interaction with LC3-II flux and turnover**. **A) **Lysates were obtained from control neuronal cultures (at 24 h), cultures treated with proteasome inhibitor lactasystin (20 μM) or with liposomal proteolysis inhibitor E64d (20 μM), or and 24 hours from neuronal cultures treated with NMDA (300 μM) or NMDA+lactacystin or E64d preincubation for 2 h. LC3-II immunoblot band intensities were quantified densitometrically and the values plotted. The expressed values are means ± S.E.M. [n = 3; *p < 0.05** p < 0.01 (Student paired T-test) compared to controls; § (p < 0.05] shows difference between NMDA+ lactacystin or E64d vs. lactacystin or E64d alone, respectively.

It is conceivable that the NMDA induced LC3-II accumulation as a result of either increased LC3-I to LC3-II conversion or the result of autophagosome (thus LC3-II) turnover inhibition. We addressed this issue with two pharmacological tools: E64d, a cell permeable lyososomal cysteine protease (cathepsin B, L) inhibitor, and lactacystin, a proteasome inhibitor that blocks cytosolic protein turnover via the ubiquitin-proteasome pathway. Our data show that lysosomal inhibition (with E64d) potently elevated LC3-II levels to 67.1 ± 1.6 densitometric units from 41.3 ± 1.6 units in controls possibly due to the inhibition of autophagosome turnover. NMDA treatment, to a lesser extent, significantly increased LC3-II levels (to 56.0 ± 1.2 units). Interestingly, under NMDA treatment conditions, the cells appear refractory to further elevation with co-treatment with E64d and lactacystin (Fig 7), suggesting that the normal autophagosome clearance pathway may no longer be in place. Thus, taken together, our data suggest that normal LC3-II turnover by lysosome as well as proteasome-pathways are inhibited by NMDA treatment.

### Cell death in NMDA-treated neurons was alleviated by 3-MA

**Figure 8:**
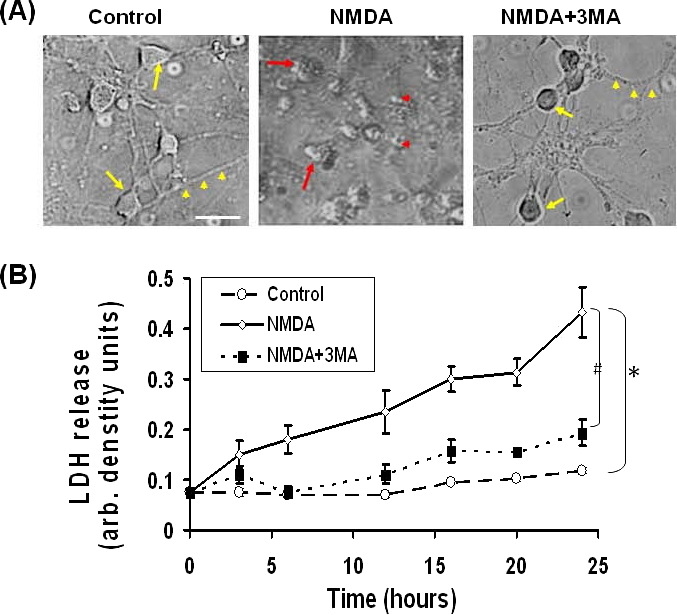
**Autophagy inhibitor 3-MA protects neurons against NMDA excitotoxcity**. **A) **Phase contrast images indicates the changes in the morphology of neurons following 16 h treatment with NMDA (200 μM) (middle panel), NMDA (200 μM) + 3-MA (10 mM) (right panel) and Control (left panel). With control CGN, arrows (yellow) indicate healthy cell body morphology while arrow heads (yellow) indicate healthy neurites. With NMDA treatment, red arrows and red arrow heads indicate degenerative neurons and apoptotic bodies, respectively. With NMDA+3 MA treatment, yellow arrow and yellow arrow heads represent relatively preserved cell bodies and neurites. Images are taken at 400× magnification. Scale bar represents 20 μm. **B) **LDH release recorded and plotted after incubating cerebellar neurons in culture in NMDA (open diamond), NMDA+3-MA (black square) and control (open circle). The expressed values are means ± S.E.M. (n = 6). *p < 0.05, ANOVA, NMDA group is significantly higher than control (Ctrl); and ^#^p < 0.05, ANOVA, NMDA+3 MA group is significantly lower than NMDA challenge group).

Neuronal cultures were divided into three treatment groups: NMDA (200 μM), NMDA+3-MA and control. Representative phase contrast images of the neurons at 16 hours following NMDA-treatment demonstrated injured and dying neurons with shrunken cell bodies and non-existing neurites when compared to healthy control CGNs. In fact, NMDA-treated neurons showed apoptotic cell morphology not seen in controls (Fig 8A). In contrast, neurons co-treated with NMDA+3-MA showed a significant sparing of neurites. While cell body shrinkage was observed, membrane integrity, however, was largely preserved. 3-MA co-treatment appears to have neuroprotective effects for cells that have been NMDA challenged. To further explore this issue, NMDA-induced cell death was assayed by measuring the lactate dehydrogenase (LDH) enzyme release into the culture medium. The LDH release increases progressively from 6 h to 24 h after NMDA-treatment while untreated control cultures have no significant increase of LDH release over the same time period (up to 24 h). Increases in LDH release following NMDA exposure was significantly alleviated when the neuronal cultures were co-treated with 3-MA. The levels of LDH release between NMDA and NMDA+3-MA co-treated cultures were significantly different at all time points between 6 hours and 24 hours (**#**p < 0.05) (Fig 8B). This confirms the neuroprotective effects of 3-MA against NMDA-mediated exictotoxicity.

### NMDA-induced caspase-3 activation is suppressed by 3-MA

**Figure 9:**
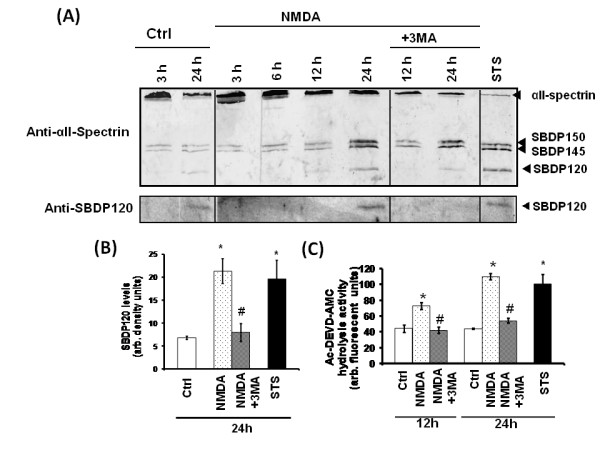
**NMDA-induced caspase-3 activation is suppressed by 3-MA**. **A) **Representative immunoblot of αII-spectrin breakdown profile shows the presence of the caspase-3 specific αII-spectrin breakdown product (SBDP) of 120 kDa in NMDA-treated cultures after 24 hours compared to the controls and NMDA+3-MA, or staursorpine (0.5 μM) (n = 3). Representative immunoblot probed with anti-SBDP120 shows a similar profile of the breakdown product in NMDA-treated cultures after 24 hours following treatment but not in controls or NMDA+3-MA co-treatment (n = 3). **B) **Densitometric analyses of the immunoblots probed with anti-SBDP120 show a significant increase in the αII-spectrin breakdown product of 120 kDa (SBDP120) after 24 hours following NMDA or STS treatment, when compared to control (*p < 0.05, paired Student T-test). NMDA+3-MA co-treatment significantly suppressed SBDP120, when compared to NMDA treatment alone (^#^p < 0.05, paired Student T-test). The expressed values are means ± S.E.M. (n = 3) **C) **Caspase-3 enzymatic assay was determined using the caspase substrate Ac-Asp-Glu-Val-Asp-7-amino-4-methylcoumarin (Ac-DEVD-AMC) incubated with protease-inhibitor free lysates obtained from cultures treated with or without NMDA and NMDA+3-MA co-treatment at 24 hours. The expressed values are means ± S.E.M. (n = 3). Caspase-3 activity in NMDA or STS treatment are significantly higher than corresponding controls (*p < 0.05, paired Student T-test). NMDA+3-MA co-treatment significantly suppressed caspase-3 activity, when compared to corresponding NMDA treatments alone (^#^p < 0.05, paired Student T-test).

In our previous study [25], we had demonstrated the activation of caspase-3 under conditions of prolonged amino acid starvation-induced autophagy in PC-12 cells. Here, we tested our hypothesis that the neuroprotective effects of 3-MA may have been achieved through caspase-3 suppression. To assess caspase-3 activation, we examined the proteolysis of an endogenous caspase-3 substrate (αII-spectrin) and caspase-3 enzymatic activity assay [11,26]. The αII-spectrin breakdown profile using total anti-αII-spectrin antibody showed an increase of the caspase-3 generated spectrin breakdown product of 120 kDa (SBDP120) at 24 h following treatment of cerebellar neurons with NMDA in culture. Increases in the calpain-generated SBDP150 and SBDP145 [17] were also observed at 24 hours with NMDA-treated cultures, suggesting the involvement of calpains (Fig 9A) as well. Staurosporine (STS) treated cultures were used as positive controls for caspase-3 activation and SBDP120 generation. To further confirm that the 120 kDa band was caspase-3 generated, immunoblots were analyzed using anti-SBDP120 specific antibody developed in-house [27]. The blots confirmed the appearance of the SBDP120 at 24 hours in the NMDA-treated cultures but not in the controls. 3-MA co-treatment suppressed the increased SBDP120 levels to near normal (Fig 9A) levels. Densitometric analysis of the immunoblots showed a significant reduction in the caspase-3 mediated SBDP120 levels in NMDA+3-MA co-treated cultures as compared to NMDA-treated cells (Fig 9B). Interestingly, calpain-mediated SBDP150 and SBDP145 were not attenuated by 3-MA. To assay the caspase-3 protease activity N-acetyl-Asp-Glu-Val-Asp-AMC (7-amino-4-methylcoumarin) (Ac-DEVD-AMC) was incubated with protease inhibitor-free cell lysates under various conditions. Caspase-3 activity was significantly increased in NMDA-treated cultures at 12 and 24 hours when compared to control cultures. On the other hand, this caspase-3 activity was significantly reduced by 3-MA co-treatment when compared to NMDA-treatment alone (12 and 24 h) (Fig 9C).

### ATG7 disruption results in neuroprotection following NMDA exposure

**Figure 10:**
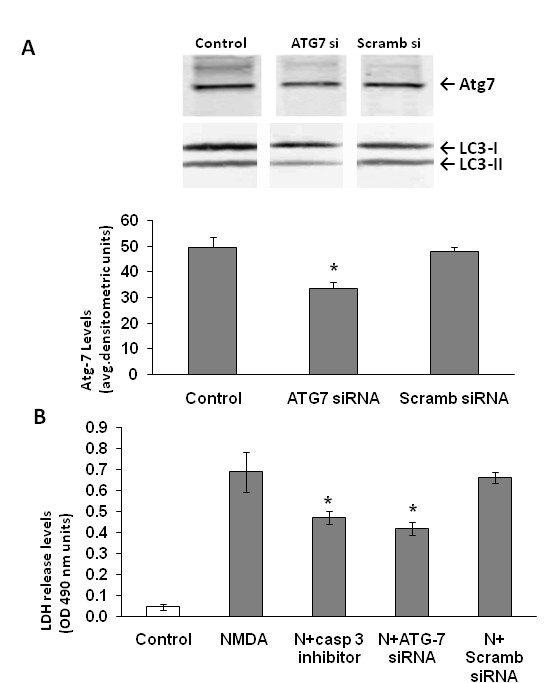
**Knockdown of ATG7 results in neuroprotection following NMDA-exposure**. **A) **ATG7 siRNA was transfected into neurons 72 hours before NMDA treatment. Representative western blot demonstrates the knockdown of the ATG7 in neurons (ATG7 siRNA) compared to controls and scrambled siRNA (Scramb siRNA). Representative LC3 immunoblot also show reduction of LC3-II band. Quantification of the band intensities (n = 3) representing the Atg7 protein levels in the granule neurons demonstrates a significant reduction in Atg 7 protein expression. Scrambled siRNA was used as a negative control to compare the efficiency of Atg7 protein suppression in neurons. *p < 0.05 ANOVA, Atg7 protein levels are significantly lower in the siRNA paradigm compared to controls and scrambled siRNA **B) **Lactate dehydrogenase (LDH) release from the neurons into the medium was measured and quantified at 6 hours post treatment (n = 3). *p < 0.05, ANOVA, NMDA+caspase-3 inhibitor (IDN- and NMDA+ATG7 siRNA treatment groups are significantly lower compared to NMDA treated neurons. Data is represented as mean ± S.E.M.

Since 3-MA might have non-autophagy related effects, ATG7 siRNA was generated and transfected into the neurons in culture to further investigate the effects of autophagy inhibition on NMDA neurotoxicity. First, we observed that atg-7 siRNA partially but significantly reduced Atg-7 protein levels as well as LC3-II levels (downstream effect) at 72 h after siRNA treatment (Fig 10A). Scrambled ATG7 siRNA (negative control-) had no effect-. We then further analyzed the effects of ATG7 siRNA and scrambled ATG7 siRNA on NMDA exposure induced cell death, as measured by LDH release assay. Silencing the Atg7 protein expression in neurons resulted in a significant (but partial) reduction of LDH release in the medium compared to neurons transfected with NMDA with scrambled ATG7 siRNA or NMDA alone. ATG7 siRNA and scrambled siRNA alone did not significantly increase LDH above control cells *(data not shown)*. Since NMDA toxicity has been demonstrated to induce apoptotic cell death, we incubated neurons with pan caspase inhibitor IDN6556 to study if we achieved neuroprotection. LDH release assay suggest comparable neuroprotection with ATG7 siRNA as well with co-treatment with pan caspase-inhibitor IDN6556 (20 μM) against NMDA mediated neurotoxicity (Fig 10B).

## Discussion and Conclusions

Autophagy induction occurs in the central nervous system under conditions of stress/starvation or protein aggregating neurodegenerative diseases [5,28-30]. This study has shown that acute excitotoxicity by NMDA exposure can act as a stressor to induce autophagy in cerebellar neurons. Glutamate excitotoxicity has previously been documented as one of the pathways of cell death following experimental traumatic brain injury [10,31,32]. Erlich and colleagues [33] demonstrated an increase in beclin-1 expression in mice following traumatic brain injury suggesting that autophagy is upregulated around the regions of injury to support the cells under duress and help dispose of damaged components. Recently, there has also been suggestive evidence for the involvement of autophagy in chronic neurodegenerative diseases such as Parkinson's disease and Huntington disease [34-36].

In our experiments we observed an increase in the autophagy protein LC3 immunostaining and the monodansylcadaverine (MDC) positive autophagosomes following NMDA treatment as compared to control samples (Figs 1, 2 and 3). The NMDA treatment also increased the levels of LC3-I when compared to the controls at earlier time periods (3 and 6 h). This transient enhancement of the LC3-I protein levels in comparison to control indicates an enhanced capability of the cells to launch an autophagic response (data not shown). There was also an increase in LC3-II levels or LC3-II/LC3-I ratio (from about 0.60 to 1.30) (Fig 5) following NMDA treatment, as measured by quantitative immunoblots. This suggests that there may be a pool of LC3-II being generated following NMDA exposure that is translocated to the outer membrane of the autophagosomes [37,38].

Evidence from other studies demonstrated the induction of autophagy and subsequent neuronal death in spinal cord motor neurons and organotypic hippocampal cultures, following glutamate receptor-mediated injury [39,40]. According to one study, a buildup of autophagosomes could be observed in the axonal terminals of neurons in Lurcher mice [41]. We extended their findings by demonstrating that NMDA in cultured neurons resulted in robust autophagosome formation throughout the cell bodies and neurites. The presence of unusually large stained autophagosomal bodies 24 hours following NMDA exposure, suggests a breakdown in the turnover machinery of the autophagosomes. Also, the presence of autophagosome accumulation in the neurons at a time when neuronal death was observed, points towards the fact that enhanced autophagy may be pushing the cells towards autophagic cell death.

The NMDA-induced L3-II accumulation could be a result of either LC3-I to LC-3-II conversion or it could signify defects in LC3-II turnover. We found that both lysosomal protease inhibition and proteasome pathway inhibition significantly elevated LC3-II protein levels compared to controls, suggesting that there are at least two pathways of LC3-II turnover. NMDA treatment conditions seem to render the cells refractory to further elevation of LC3-II protein levels with co-treatment with E64d and surprisingly with lactacystin. The significance of proteasome involvement in LC3-II clearance will need further investigation. Our data suggest the proteasome system is a novel, alternative pathway for clearance of LC3-II [either membrane-associated pool or non-membrane-associated associated (cytosolic) pool] under conditions of stress.

Since prolonged autophagy has been shown to result in autophagic cell death (type II), we hypothesized that this form of cell death may be a crucial component to NMDA excitotoxicity. To test this hypothesis we employed the autophagy inhibitor 3-MA and examined whether autophagy inhibition could alleviate NMDA-mediated neuronal death. The results showed effective inhibition of the NMDA-induced increase of the lipidated LC3-II protein, the latter being important in the stabilization of the autophagosomal membrane. Also, a strong decrease in the LC3 immunostaining and the MDC-positive autophagosome staining with 3-MA co-treatment following NMDA exposure were observed as well. 3-MA co-treatment in neurons [45-47] also resulted in significant protection against NMDA-induced cell death (Fig 8). To avoid complete reliance on interpretation of 3-MA treatment effects, we used the siRNA approach to investigate the role of autophagy in NMDA mediated neurotoxicity [47]. Employing ATG7 siRNA significantly decreased LDH release (a marker for compromised cell membrane integrity) from neurons into the culture medium, suggesting its neuroprotective effects (Fig 10).

Apoptotic and autophagy pathways are intricately intertwined in the cell [42-44]. In our experiments we observed that NMDA-induced caspase-3 enzyme activation and breakdown of spectrin were 3-MA sensitive. Also, intervention with the pan caspase inhibitor IDN-6556 (20 μM) resulted in partial but significant protection against NMDA-mediated excitotoxicity, suggesting the involvement of other cell death pathways. These observations suggest that autophagy and caspase-3 activation may be interlinked.

In summary, based on several lines of evidence, this study shows that autophagosomes accumulated in neurons when subjected to direct excitotoxic NMDA exposure in a simple culture paradigm. Secondly, based on (i) the presence of MDC- and LC3-densely stained autophagosomes following NMDA treatment (but not amino acid starvation paradigm) and (ii) the refractory response of LC3-II to E64d treatment in the presence of NMDA, it appears that NMDA exposure impairs or diverts the LC3-II/autophagosomes from normal clearance. Autophagy inhibition via 3-MA and ATG7 siRNA provides neuroprotection against NMDA toxicity.

Taken together, we propose that NMDA neurotoxic stress triggers the neurons into an autophagic response. Impairment in autophagosomes clearance causes the neurons to be 'stuck" in the early autophagosome accumulation mode, which appears to be detrimental to cell survival. In other words, autophagy under NMDA toxicity paradigm in fact contributes to the neuronal cell injury/cell death process. Obviously, more research into this direction will be needed to confirm this hypothesis. It is also equally important to examine how widespread this autophagic response phenomenon is in other neurotoxic conditions either in culture or *in vivo*.

## Methods

### Chemicals and Antibodies

N-methyl D aspartate (NMDA), 3-methyladenine (MA), monodansylcadaverine (MDC), lysosomal protease inhibitor E64D and proteasome blocker lactacystin were purchased from Sigma Laboratories (St. Louis, MO). Prolong Antifade was purchased from Molecular Probes (Eugene, OR). Fetal bovine serum and Dulbecco's modified eagle's medium (DMEM) was from Gibco laboratories (Grand Island, NY). Antibodies mouse monoclonal anti-αII-spectrin and rabbit polyclonal anti-caspase-3 specific spectrin breakdown product of 120 kDa (SBDP120) [26,27] were made in-house as was pan caspase inhibitor IDN-6556 [49]. Anti-β-actin antibody was purchased from Sigma laboratories (St.Louis, MO), anti-NeuN antibody was obtained from Chemicon Laboratories (Temecula, CA) and anti-LC3 antibody from Novus Biologicals (Littleton, CO).

### Animals

Female pregnant Sprague Dawley rats (Harlan Laboratories) were received and housed in individual cages. The animals were maintained on a 12 h light/dark cycle with food and water ad libitum. All the experimental procedures in the animals were performed in accordance with the National Institute of Health Guide for the Care and Use of Laboratory Animals and the protocols were approved by the UF IACUC.

### Cell cultures and treatments

Cerebellar cultures were obtained from dissociated cerebella of 6-8 day old Sprague Dawley rat pups (Harlan Laboratories) and plated in Dulbecco's modified eagle's medium (DMEM) supplemented with 25 mM glucose, 25 mM KCl, 10% fetal bovine serum on culture dishes (Nunc plates, Fisher). 1β-arabinofuranosylcytosine (10 μM) was added to the culture medium 22 hours after plating to prevent the proliferation on non-neuronal cells for 48 hours. On the 8^th ^day following harvesting, the neurons were exposed to different treatment conditions and subsequent experimental end points. The neurons were treated with or without NMDA (200 μM) for different time periods and the cells were eventually lysed with triton based lysis buffer for protein immunoblots. The other treatment condition involved a co-treatment of NMDA with 3-methyladenine (3-MA, 10 mM). For fluorescent microscopy, the neurons were cultured on glass coverslips coated with poly-l-lysine and treated in a similar manner as the cultures on plates. Positive control to induce autophagy involved subjecting CGNs to amino acid starvation by incubating them in Hank's buffer with 25 mM glucose (with calcium, magnesium) and vitamins.

### Immunofluorescence

Cerebellar cells plated on coverslips were fixed using freshly prepared 4% paraformaldehyde solution for 10 min at 4°C, washed in pure methanol and then permeabilized with 1× Tris buffered saline -Tween-20 (TBST, Sigma Laboratories, St. Louis, MO). Following TBST washing, the cells were incubated in 5% normal goat serum (NGS) at 37°C for 30 minutes before incubating with the primary antibody microtubule associated light chain-3 (LC-3; Atg8; 1:1000) in 5% NGS overnight at 4°C. On the following day, the coverslips were washed 3 times with 1× TBST and incubated with the Alexa Fluor (Molecular Probes, Carlsbad, CA) red or green-conjugated secondary antibodies (1:3000) for 1 hour at 37°C. The coverslips were then washed with 1× TBST and then mounted with the mounting medium Vectashield (Burlingame, CA) and observed under the microscope.

### Autophagosome labeling with MDC

The neurons on the coverslips were incubated with a fluorescent dye monodansylcadaverine (MDC; 0.05 mM) in phosphate buffered saline (PBS) after 6, 12, 16 and 24 hour NMDA challenges at 37°C for 10 minutes, to observe for autophagosomes [50]. The cells were washed 2 times with PBS, mounted using Antifade solution (Prolong Antifade) and immediately observed by Zeiss fluorescence microscope. Following prolonged NMDA, we observed unusually large autophagosomal bodies, which are defined as having a size at least 5-time the size of normal sized autophagosomes. For their quantification of both average sized autophagosomes and unusually large autophagosomal bodies, for each experimental condition/well, 4 randomly selected fields per well (400× magnification) are used and more than 20 cells in each field are analyzed with counted.

### Western Blot Analysis

The cerebellar neuronal lysates were collected at different time points after treatment with the appropriate media using lysis buffer containing 1% (v/v) Triton X-100, 5 mM EGTA, 5 mM EDTA, 150 mM NaCl and 20 mM Tris HCl (pH 7.4). The protein content was determined using DC Protein Assay (Bio-Rad, Hercules, CA) and the protein concentration was standardized to 1 μg/μL. Twenty micrograms of protein were subjected to SDS-PAGE gel electrophoresis on 4-20% or 6% Tris-glycine gels (Invitrogen, Carlsbad, CA) and then transferred onto PVDF membrane on a semi-dry electro transferring unit (Bio-Rad). Following the transfer, the membranes were blocked in 5% nonfat dry milk in 1× Tris buffered saline with Tween-20 (TBST) and probed overnight with primary antibody at 4°C. The following day, the membranes were washed with TBST and probed with either secondary peroxidase conjugated anti-rabbit or the biotinylated anti-mouse antibody. Immunoreactivity was detected by either using streptavidin alkaline phosphatase conjugate tertiary antibody or enhanced chemiluminescence (ECL) reaction (Amersham, Piscataway, NJ). Densitometric quantification of the bands was performed using ImageJ software (version 1.29×; NIH, Bethesda, MD).

### Lactate Dehydrogenase (LDH) Release Assay for Cell Death

Lactate dehydrogenase release assay was performed to assess cell death by measuring the release of lactate dehydrogenase into the medium from damaged cells due to necrosis and secondary necrosis following apoptosis [51] or autophagic cell death. Culture medium, 25 μL, was collected after 0, 3, 6, 12, 16, 20 hours and 1 day in 96-well flat bottom plates. An equivalent volume (25 μL) of detection reagent (CytoTox One Reagent, Promega, Madison, WI), was added to each well containing the culture medium and incubated for 30 minutes in the dark at room temperature. Absorbance was measured (wavelength: 490 nm) using a colorimetric microplate reader (SpectraMax Gemini, Molecular Devices). Six replicates for each time point per experiment were assayed and three such experiments were performed. The arbitrary density unit values were plotted against time.

### Caspase-3 enzymatic activity assay

To assay for caspase-3 activity, control, NMDA-treated and NMDA/3-MA co-treated granule neurons from three different wells (12 and 24 h) were scraped in a buffer containing 20 mM Tris-HC1 (pH 7.4 at 4°C), 150 mM NaC1, 1 mM dithiothreitol, 5 mM EDTA, 5 mM EGTA, and 1% (vol/vol) Triton X-100 for 1 h. The cleared lysates were mixed with 50% (vol/vol) glycerol. Cell lysates were assayed with 100 μM acetyl-Asp-Glu-Val-Asp-7-amido-4-methylcoumarin (Ac-DEVD-MCA; Bachem Bioscience), 100 mM HEPES, 10% glycerol, 1 mM EDTA, 10 mM dithiothreitol. Fluorescence (excitation, 380 ± 15 nm; emission, 460 ± 15 nm) was measured at 60 minutes using a fluorescent microplate reader (SpectraMax Gemini EM, Molecular Devices) as described previously [52].

### ATG7 short interfering RNA (siRNA) preparation and transfection

Small interfering RNA (siRNA) targeting rat *ATG7 *(5'-GCAUCAUCUUUGAAGUGAA-3'; Sigma), or pooled non-targeting control siRNA (5'-AUGAACGUGAAUUGCUCAA-3', 5'-UAAGGCUAUGAAGAGUAC-3', 5'-AUGUAUUGGCCUGUAUUAG-3', and 5'-UAGCGACUAAACACAUCAA-3'; Dharmacon) [53] were purchased commercially and used. Primary neurons were transfected on day 4 of culture preparation, with 20 nmol of *ATG7 *or control siRNA using Lipofectamine 2000 (Invitrogen), 72 hours before subjecting to NMDA challenge. The efficacy of ATG7 knockdown was assessed by Western blot using an antibody against Atg7 (Sigma).

### Statistical Analysis

One-way ANOVA with Tukey post hoc test was used to draw comparisons between groups in the LDH assay. Data was plotted as means ± S.E.M. (standard error of the mean). A value of p ≤ 0.05 was considered to be significant. Student's t-test was performed to draw statistical comparisons between two treatment groups and a p < 0.05 was considered to be statistically significant.

## Abbreviations

3 MA: 3-methyladenine, MDC: monodansylcadaverine, MAP-LC3: microtubule associated protein-light chain 3.

## Authors' contributions

SS and KKW planned, designed and conducted experiments. SS, KKW and RLH wrote the manuscript. RLH, SFL, ZZ, FHK, MCL and WZ helped perform some of the experiments, interpretation of data and revise aspects of the manuscript. All authors have read and approved the final version of the manuscript.
